# Pharmacodynamic Properties of Subcutaneous Immunoglobulin in Myasthenia Gravis: Sub-analyses From an Open-Label Trial

**DOI:** 10.3389/fneur.2020.00921

**Published:** 2020-08-25

**Authors:** Brendan N. Putko, Grayson Beecher, Zaeem A. Siddiqi

**Affiliations:** Division of Neurology, Department of Medicine, University of Alberta, Edmonton, AB, Canada

**Keywords:** subcutaneous immunoglobulin, pharmacodynamics, myasthenia gravis, hizentra, serum level

## Abstract

**Background:** We previously reported an open-label prospective trial of subcutaneous immunoglobulin (SCIg) in mild to moderate exacerbations of myasthenia gravis (MG). The effective dose of SCIg in MG and whether measured immunoglobulin G (IgG) levels correlated with measures of disease burden were not reported.

**Objectives:** To understand the relationship between SCIg dosing and serum IgG levels on measures of disease burden: quantitative MG (QMG), MG activities of daily living (MG-ADL), MG composite (MGC), and manual muscle testing (MMT) scores.

**Methods:** We performed *post-hoc* analyses of variance to assess change in oculobulbar and generalized sub-scores. We assessed the improvement in QMG, MG-ADL, MGC, or MMT over intervals from baseline to week 2, weeks 2–4, and week 4 to end of study. Improvement was either greater than (coded 1) or was equal to or less than (coded 0) the previous 2 weeks. Binaries were assessed in binary logistic regression as a function of SCIg dose over the two-week interval as the independent variable. We also performed linear regression analyses with change in the clinical scores as the dependent variable and change in IgG level over the entire study period and over the interval from weeks 2 to 4, during which change in IgG level was maximal, as the independent variables.

**Results:** Subanalysis of QMG and MG-ADL scores demonstrated significant reductions in the oculobulbar and the generalized portions of both measures. Binary logistic regression analyses did not find any statistically significant correlations between the odds of improvement and weight-adjusted dose of SCIg over 2-week intervals. There were no significant relationships between changes in scores and IgG level over the entire study period or over the interval from weeks 2 to 4.

**Conclusions:** Although SCIg dose varied over the study period, the odds of improvement were not significantly correlated with this, which suggests that the current dose of 2 g/kg for SCIg should be compared to different, possibly lower, dosing regimens head-to-head. The change in clinical scores was not significantly associated with IgG levels suggesting a complex relationship. SCIg may be effective for both ocular and generalized presentations of MG.

## Introduction

Autoimmune myasthenia gravis (MG) is a disorder of the post-synaptic neuromuscular junction characterized by fluctuating, fatigable weakness that can affect extraocular, bulbar, limb, and respiratory muscles (1). Immunomodulatory therapy is a central pillar in the treatment of autoimmune MG. Preparations of exogenous human immunoglobulin, which include subcutaneous immunoglobulin (SCIg) and intravenous immunoglobulin (IVIg), have been used in various autoimmune disorders, but the role of SCIg in treating neuromuscular disorders was defined in chronic inflammatory demyelinating polyradiculoneuropathy, while the data in MG are limited but encouraging (2). We previously reported the results of an open-label prospective trial of SCIg 2 g/kg total in mild to moderate exacerbations of MG that was conducted over 6 weeks with assessments at baseline, weeks 2, 4, and 6 (end of study) wherein we demonstrated that SCIg is effective, safe, and tolerable (3). A case report demonstrated stabilization and maintenance with SCIg alone in one of two patients with MG (4), while a growing body of evidence supports the efficacy of SCIg in maintenance of stable MG (2, 5–8).

Reported below are the results of *post-hoc* analyses that we conducted on data collected for our previously reported open-label prospective trial (3). The rationale for further analyses are as follows: we observed continued improvement over the study period; however, there was a robust early response, which we hypothesized would meet thresholds of clinically meaningful responses in the quantitative MG (QMG) (9), MG activities of daily living (MG-ADL) (10), and MG composite (MGC) (11) scores. We also sought to assess the effect of SCIg on oculobulbar and generalized manifestations of MG, which are captured by portions of the QMG (12) and MG-ADL (13). Furthermore, while the total dose in our study was 2 g/kg, we dosed the study drug in a dose-escalating manner (3), such that the weekly interval dose varied across the study. We therefore analyzed whether the dose was associated with the rate of improvement in the aforementioned clinical scores along with manual muscle testing (MMT) score, as the effective dose for SCIg has not been defined and the current recommendations have been generated by extrapolation from other conditions and from IVIg dosing (2). Also, one of the drawbacks of SCIg as compared to IVIg is the time required to infuse the full dose (2 g/kg), which may not be practical in MG exacerbations where rapid treatment is required. The safety and tolerability of SCIg demonstrated in the trial results suggests that the dose may be given over a shorter period (3). It is plausible that faster infusion of SCIg may result in earlier peak in the clinical response. To that end, we undertook the analysis to assess the impact of the rate of change of serum IgG levels on the clinical parameters.

## Methods

Full methodology concerning the recruitment and assessment of trial participants is described in our original report (3). In summary, this was a phase 3, open-label, prospective trial with a single study arm that included a total of 22 participants who successfully completed the trial. Of the 22 that completed the trial, three had a subsequent exacerbation and were re-enrolled, such that we studied 25 instances of MG exacerbation treated with SCIg in addition to other standard therapies. MG exacerbation was defined as transitioning of a patient to a higher class as per Myasthenia Gravis Foundation of America (MGFA) clinical classification i.e., from Class I to II or III, or from Class II to III.

All statistical analyses were completed using SPSS version 26. We performed *post-hoc* responder analyses using assessments from study weeks 2, 4, and 6 (end of study). Based on previous reports, a clinically meaningful response was defined as ≥3-point improvement from baseline to week 6 for QMG (9, 14), MG-ADL (10), and MGC (11). Additionally, a more stringent ≥5-point cut-off was applied for QMG (15). We analyzed sub-scores for oculobulbar and generalized weakness for QMG (12) and MG-ADL (13). Sub-score analyses were performed with one-way repeated measures analysis of variance where Mauchly's test was used to assess sphericity. In cases where sphericity was not met, the Greenhouse-Geisser correction was used for ε <0.75 and the Huynh-Feldt correction was used for ε > 0.75. The results of statistical analyses are reported in-text and graphical representations in the form of line and dot plots are presented as figures.

Our protocol was that of flexible dose-escalation based on body weight and patient tolerability (3), and participants administered their weekly dose divided over multiple days. As a result of participants starting their study drug mid-week, their dosing schedules were not necessarily aligned with their assessment days, which occurred on set weekdays. We thus calculated dose totals at the three study assessment points: week 2, week 4, and end of study (week 6). Peak dosing occurred during the interval from weeks 2 to 4. We analyzed whether the dose of SCIg during the three assessment intervals—baseline to week 2, weeks 2–4, and week 4 to study end—correlated with the change in the four clinical scores studied, QMG, MG-ADL, MGC, and MMT. We explored the question as a binary whereby the answer was that improvement in a given score was greater (coded 1), or that it was equal to or less than (including no improvement or worsening; coded 0) as compared to the previous 2-week interval. We thereafter performed binary logistic regression analyses where the independent variable was SCIg dose over the 2-week interval in question expressed in g/kg and the dependent variable was the binary for score improvement. The results of statistical analyses are reported in-text and graphical representations in the form of binary fitted plots are presented as figures.

We performed univariate linear regression analyses where the independent variables were the change in IgG level from baseline to end of study and change in IgG level from weeks 2 to 4, and the dependent variables were the changes in clinical scores over the same intervals. The results of statistical analyses are reported in-text and graphical representations in the form of scatter plots with lines of best fit generated using the least-squares method are presented as figures.

## Results

**Figure 1:**
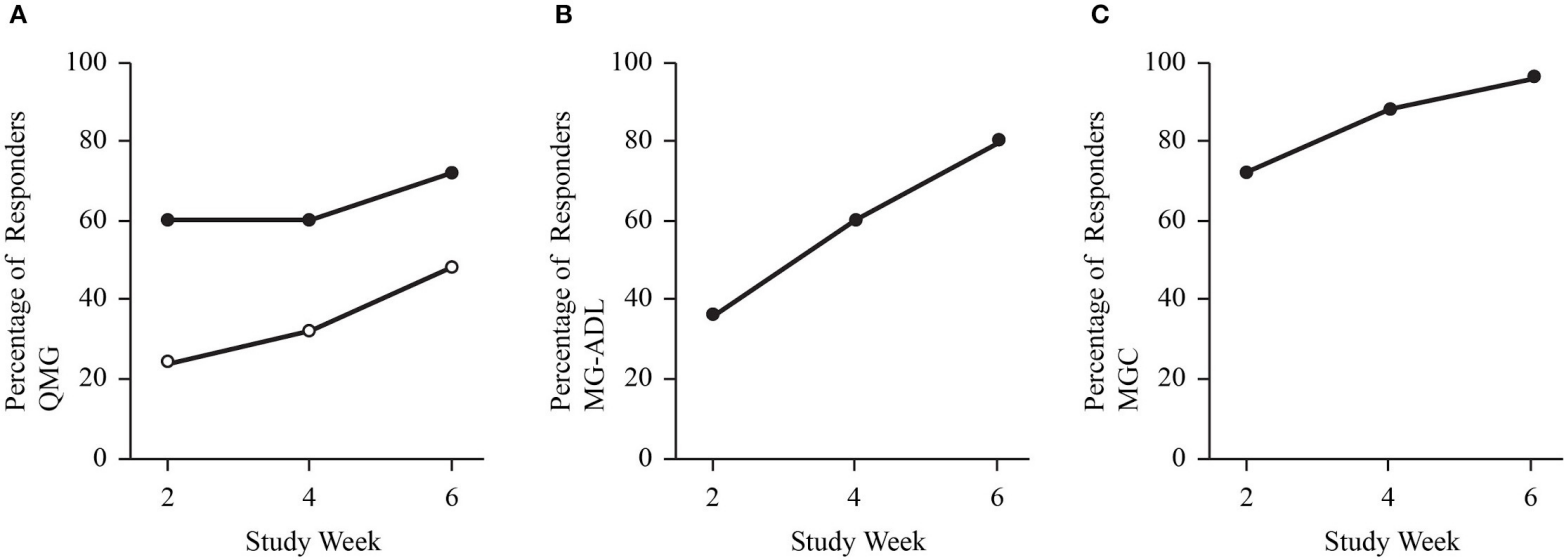
Dot plots demonstrate the proportion of study participants with a clinically meaningful improvement in QMG **(A)**, MG-ADL **(B)**, and MGC score **(C)**. Solid dots represent a cut off of 3-or-more point improvement. Open dots represent a cut off of 5-or-more point improvement for QMG.

**Figure 2:**
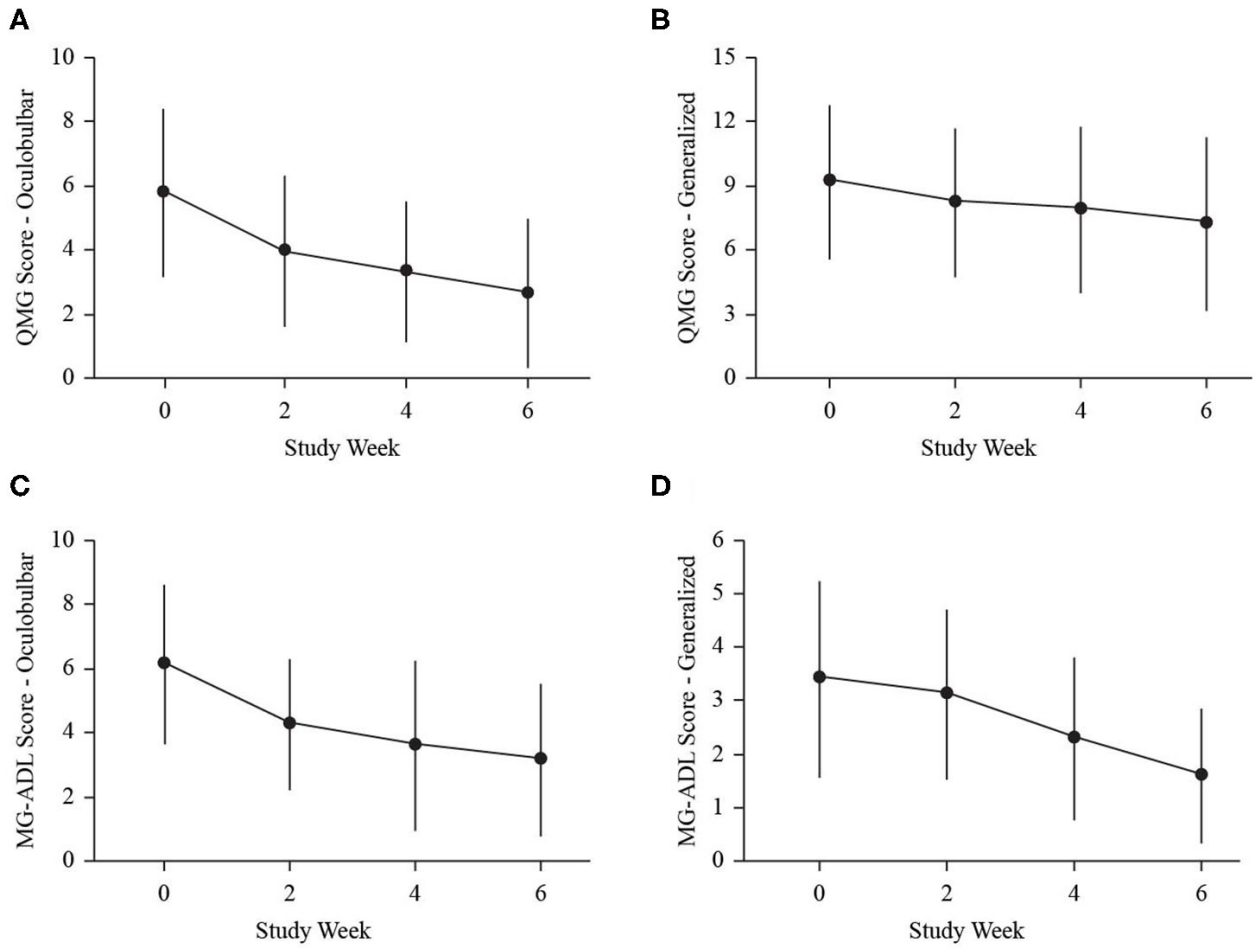
Dot and line plots demonstrate change in mean clinical sub-scores: oculobulbar QMG **(A)**, generalized QMG **(B)**, oculobulbar MG-ADL **(C)**, and generalized MG-ADL **(D)** at baseline (0), week 2 (2), week 4 (4), and end of study (6). Error bars represent standard deviation.

The mean dose from baseline to week 2 was 0.47 ± 0.15 g/kg (range 0.26–0.96 g/kg), and the mean dose from weeks 2 to 4 was 1.07 ± 0.14 g/kg (range 0.79–1.39 g/kg). In 22 exacerbations (88%), the dosing was completed after the week 4 assessment, such that the mean dose after week 4 was 0.35 ± 0.14 g/kg (range 0–0.55 g/kg). The proportion of individuals with a clinically meaningful response increased at each assessment (weeks 2, 4, and end of study; Figure 1). At study end, 72% (3-point) and 48% (5-point) of exacerbations for QMG (Figure 1A), 80% for ADL (Figure 1B), and 96% for MGC (Figure 1C) had a clinically meaningful response. The maximum improvements in QMG, MG-ADL, MGC, and MMT were 12 points, 11 points, 22 points, and 45 points, respectively. Sub-score analysis of QMG and MG-ADL scores demonstrated statistically significant reductions in the oculobulbar portion of QMG [F_(3, 72)_ = 17.92, *p* < 0.001, Figure 2A] and MG-ADL [F_(2.23, 53.44)_ = 23.94, *p* < 0.001, Figure 2C], as well as the generalized portion of QMG [F_(1.83, 43.84)_ = 5.43, *p* = 0.009, Figure 2B] and MG-ADL [F_(2.17, 51.96)_ = 23.25, *p* < 0.001, Figure 2D].

**Figure 3:**
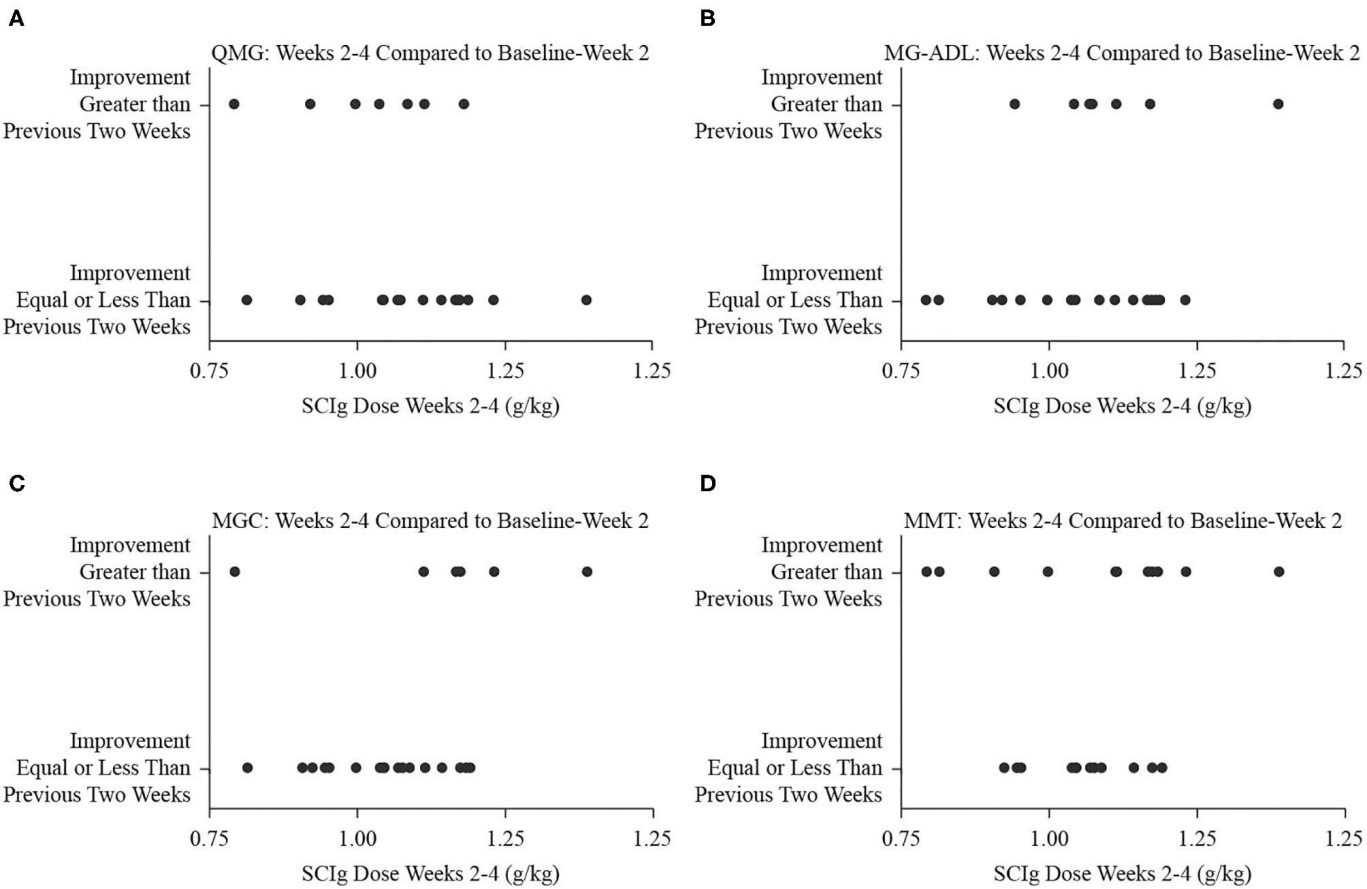
Binary fitted plots comparing the improvements in QMG **(A)**, MG-ADL **(B)**, MGC **(C)**, and MMT **(D)** scores over the interval from weeks 2 to 4 to the interval from baseline to week 2. The binary answer was that improvement was greater, or that it was equal to or less than, including no improvement or worsening. These results (dependent variable) were plotted on the SCIg dose over the interval from weeks 2 to 4 adjusted for patient weight (independent variable).

**Figure 4:**
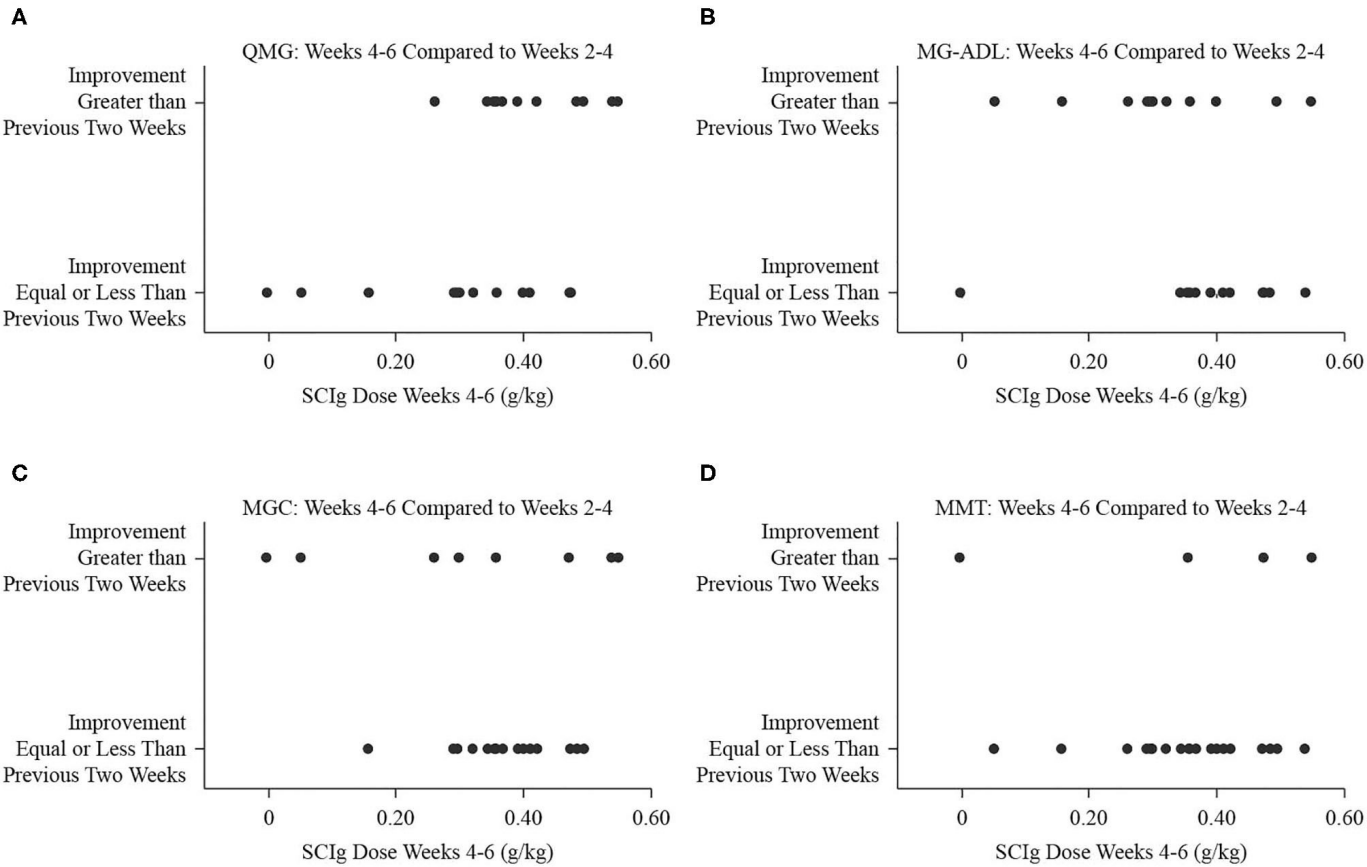
Binary fitted plots comparing the improvements in QMG **(A)**, MG-ADL **(B)**, MGC **(C)**, and MMT **(D)** scores over the interval from weeks 4 to 6 (end of study) to the interval from weeks 2 to 4. The binary answer was that improvement was greater, or that it was equal to or less than, including no improvement or worsening. These results (dependent variable) were plotted on the SCIg dose over the interval from weeks 4 to 6 adjusted for patient weight (independent variable).

Comparing the study period from weeks 2 to 4 to the period from baseline to week 2, the number of participants who experienced greater degree of improvement was 8 (32%) for QMG, 9 (36%) for MG-ADL, 7 (28%) for MGC, and 12 (48%) for MMT. The dose of SCIg from weeks 2 to 4 was not significantly associated with the odds of improvement for any of QMG (B = −3.97, *p* = 0.268, Figure 3A), MG-ADL (B = 3.95, *p* = 0.283, Figure 3B), MGC (B = 6.57, *p* = 0.138, Figure 3C), or MMT (B = 1.27, *p* = 0.684, Figure 3D). Comparing the interval from weeks 4 to 6 (study end) to the interval from weeks 2 to 4, the number of participants who experienced greater degree of improvement was 12 (48%) for QMG, 11 (44%) for MG-ADL, 8 (32%) for MGC, and 5 (20%) for MMT. The dose from week 4 to end of study was not significantly associated with the odds of improvement for any of QMG (B = 9.28, *p* = 0.065, Figure 4A), MG-ADL (B = −3.97, *p* = 0.250, Figure 4B), MGC (B = −2.95, *p* = 0.366, Figure 4C), or MMT (B = −0.52, *p* = 0.897, Figure 4D).

**Figure 5:**
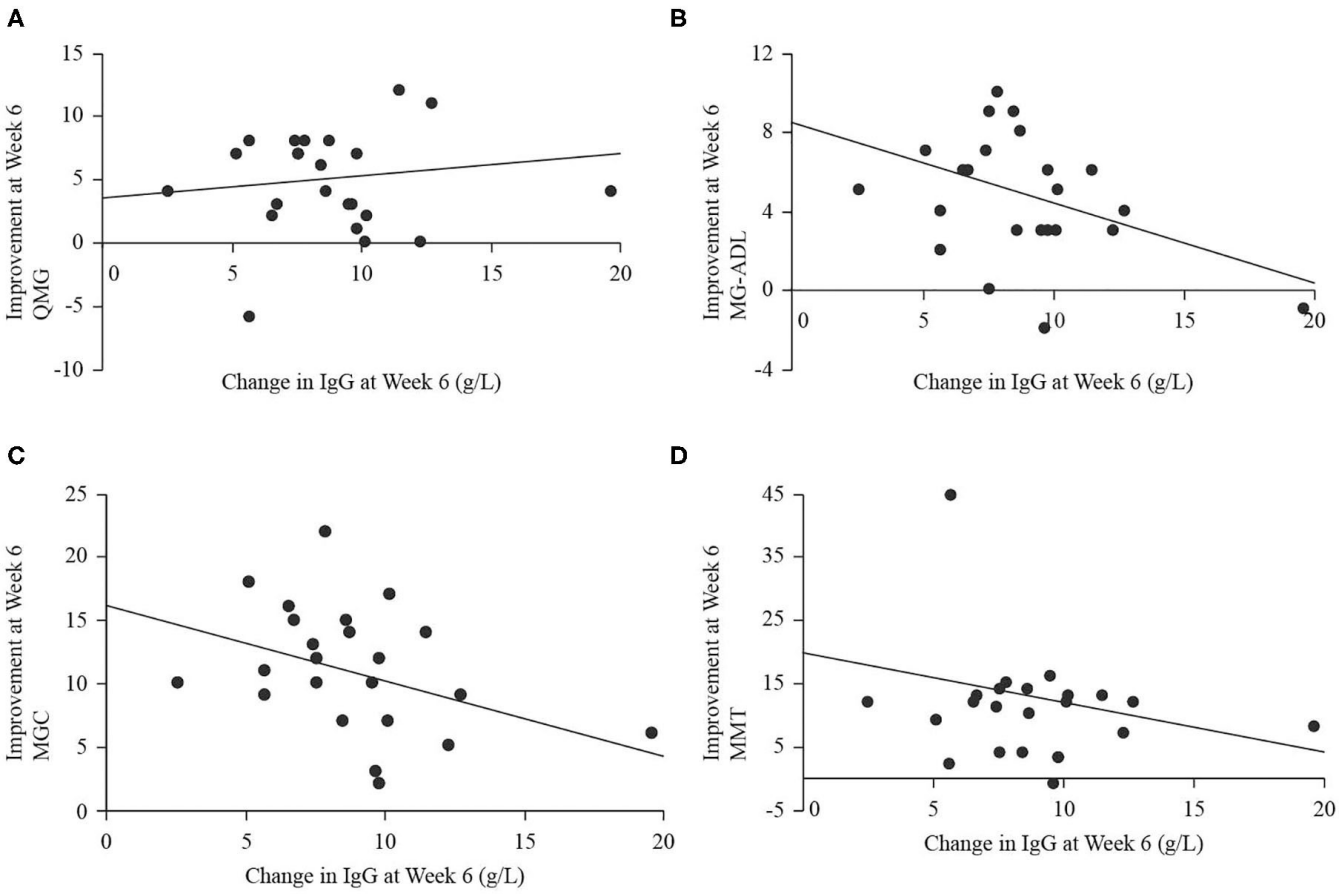
Scatter plots with regression lines for improvement in QMG **(A)**, MG-ADL **(B)**, MGC **(C)**, and MMT **(D)** from baseline to week 6 (end of study; dependent variables) plotted on change in IgG level from baseline to week 6 (independent variable).

**Figure 6:**
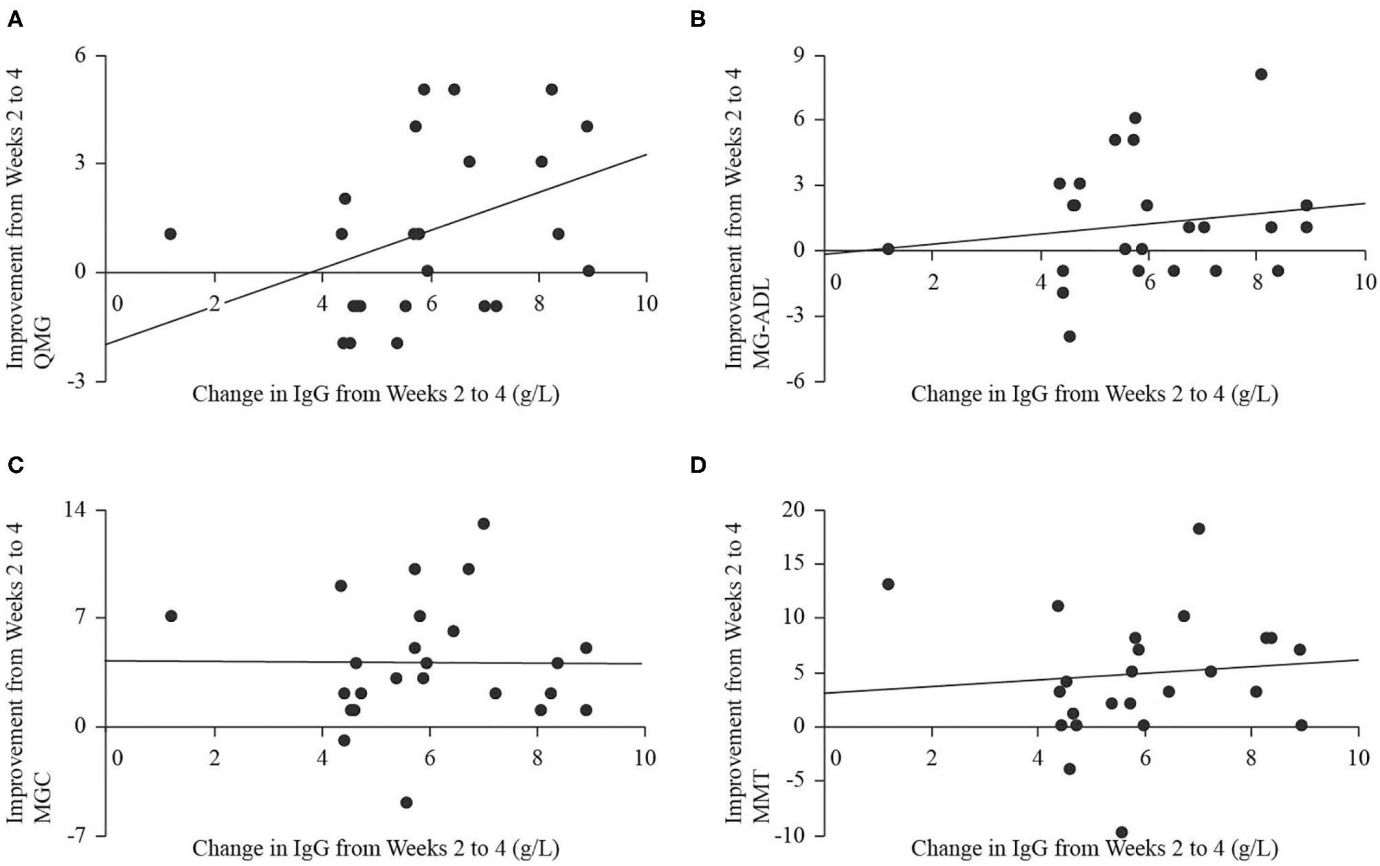
Scatter plots with regression lines for improvement in QMG **(A)**, MG-ADL **(B)**, MGC **(C)**, and MMT **(D)** from weeks 2 to 4 (dependent variables) plotted on change in IgG level from weeks 2 to 4 (independent variable).

Serum IgG levels significantly increased at end of study (18.3 ± 3.6 g/L) compared to baseline [9.3 ± 2.3 g/L, *t*_(22)_ = 12.74, *p* < 0.001], and the largest change occurred over the period from weeks 2 to 4, when IgG levels increased by 6.0 ± 1.8 g/L. There were no significant relationships between the magnitude of change in IgG level from baseline to end of study (independent) and improvement in any of QMG (dependent, B = 0.09, *p* = 0.746, Figure 5A), MG-ADL (dependent, B = −0.37, *p* = 0.064, Figure 5B), MGC (dependent, B = −0.54, *p* = 0.085, Figure 5C), or MMT (dependent, B = −0.56, *p* = 0.334, Figure 5D). There were no significant relationships between the magnitude of change in IgG level from weeks 2 to 4 (independent) and improvement in any of QMG (dependent, B = 0.47, *p* = 0.088, Figure 6A), MG-ADL (dependent, B = 0.23, *p* = 0.477, Figure 6B), MGC (dependent, B = −0.29, *p* = 0.951, Figure 6C), or MMT (dependent, B = 0.18, *p* = 0.795, Figure 6D).

## Discussion

We performed *post-hoc* analyses that demonstrated continued improvement over the study period, despite peak SCIg dosing occurring in the middle of the study. We also found statistically significant reductions in the oculobulbar and generalized sub-scores of both the QMG and MG-ADL. Several of our patients were already treated with prednisone, and this study did not compare SCIg to other treatment modalities. Previous evidence has implicated a differential whereby prednisone was more effective for ocular manifestations, and IVIg or therapeutic plasma exchange were more effective for generalized manifestations (16). Our data are encouraging that SCIg may be used as a therapy in various presentations of MG.

Binary logistic regression analyses did not find a relationship between the dose, expressed in g/kg, and the odds of improving more or less relative to a previous 2-week interval. When comparing the interval from baseline to week 2 to the interval from weeks 2 to 4, increasing the dose of SCIg did not portend further improvement. Similarly, as the dose tapered in the interval from week 4 to end of study, the odds of improvement, or lack thereof, did not significantly correlate with the dose. Extrapolating the doses in the intervals from baseline to week 2 or from week 4 to end of study over three dosing intervals yields total doses below 2 g/kg (1.41 g/kg for weeks 2–4 and 1.05 g/kg for week 4 to end of study, respectively), suggesting that lower doses of SCIg may be sufficient to both initiate and maintain a response in patients with mild to moderate MG exacerbations. In view of the large volumes required and the resultant time over which 2 g/kg needs to be administered, head-to-head comparisons of 1 g/kg and 2 g/kg regimens are warranted. To date, this has not been formally evaluated in MG for SCIg *per se*. A single reported case achieved stabilization of MG with 0.16 g/kg per week (4). In looking beyond SCIg in MG, there is no evidence of a difference in efficacy between 1 and 2 g/kg dosing regimens for IVIg in MG exacerbation (17). Furthermore, a phase 3 trial in maintenance treatment of chronic inflammatory demyelinating polyradiculoneuropathy with SCIg that compared high dose (0.4 g/kg per week), low dose (0.2 g/kg per week), and placebo found significantly less relapses in the treatments groups compared to placebo, but no significant difference between treatment groups (18). Beyond the obvious benefit of lower doses conferring a lower likelihood of side effects, there remains doubt about the role of SCIg in severe exacerbations and crises given the infusion volumes and infusion times required (5), which could potentially be ameliorated by lower dosing requirements.

The regression analyses we performed did not demonstrate significant correlations between clinical scores and the change in IgG level, although there was a trend toward significance for negative correlations between improvement in MG-ADL and change in IgG level as well as improvement in MGC and change in IgG level over the entire study period. There was also a trend toward significance for a positive relationship between improvement in QMG and change in IgG level over the interval from weeks 2 to 4. Without a larger data set to explore whether these relationships would achieve significance, their meaning should not be overstated. In view of previous reports of stable IgG titers in patients treated with SCIg without the peak and trough changes expected from IVIg (5), it is possible that a steady state IgG level, manifested in minimal change from baseline, indicates effective immunomodulation and a resultant improvement in measures of disease burden. Indeed, we showed that peak IgG levels occurred before study end (3), but did not have longer term follow up data to assess whether levels remained elevated near the peak level or declined to a lower steady state. The trend toward significance for the positive relationship between QMG and IgG over the interval from weeks 2 to 4, during which change in IgG level was maximal, may represent attenuation of autoimmunity during rapid IgG rise. Taken together, a rapid rise in IgG may be required to blunt the immune response, then a steady state may be required to maintain relative quiescence. Adding to the complexity of using IgG levels as biomarker is the evident relationship between IgG levels and outcomes in acute demyelinating neuropathies, and lack of evidence for the same relationship in chronic demyelinating neuropathies (19). Clearly, more research is required to define the physiologic changes associated with SCIg infusion in MG, and subsequently to assess whether serum IgG titers have a role as a biomarker of immunomodulation in this condition. Finally, therapeutic mechanisms of SCIg are not fully understood, though evidence exists to support a pleotropic immunomodulatory role that extends beyond IgG levels and into other components of humoral as well as cellular immunity (20).

Considering that we demonstrated early and continued clinically meaningful improvement in all the disease scores evaluated, there is rationale for a strategy that employs sustained dosing in order to provide patients an adequate therapeutic trial before discontinuing SCIg. While our trial did not include long-term follow-up data, others have shown SCIg is a viable maintenance therapy in MG (2, 5–8). Adding to this, it is clear that SCIg is safe and tolerable, which lends further support to providing an adequate therapeutic trial for a given patient.

Our initial report indicated that mild adverse reactions occurred, but no serious adverse reactions, including hemolysis or acute kidney injury, were observed (3).

## Limitations

The primary limitation of our study is that it was an open-label trial without a control arm wherein all participants followed a dose-escalation protocol. The analyses we presented regarding dosage and clinical improvement in Figures 2, 3 were made possible by the variability in per interval doses that occurred as a result of differences in individual tolerances and the interface of dosage timing and assessment days. As such, a secondary limitation is that the *post-hoc* analyses we present support the need for further investigation, but the trial was not specifically designed to answer a question regarding different dosing regimens.

## Conclusions

Although the dose of SCIg varied over our study period, the odds of improvement were not significantly correlated with this, which suggests that lower doses of SCIg may be sufficient to both initiate and maintain a clinically meaningful response in patients with mild to moderate MG exacerbations. Head-to-head comparisons of different, possibly lower, dosing regimens for SCIg are warranted. The change in clinical scores was also not significantly associated with IgG levels, but there was a trend toward a negative relationship over the entire study period whereby less improvement in MG-ADL and MGC was seen with larger changes in IgG, but there was also a trend toward a positive relationship over weeks 2 to 4 for QMG and IgG level. These findings require further investigation; however, they indicate the complex nature of the effects of SCIg administration.

## Data Availability Statement

The raw data supporting the conclusions of this article will be made available by the authors, without undue reservation.

## Ethics Statement

The studies involving human participants were reviewed and approved by HREB, University of Alberta. The patients/participants provided their written informed consent to participate in this study.

## Author Contributions

BP performed the statistical analyses and drafted the manuscript. GB performed initial data collection and revised the manuscript. ZS designed and supervised the original study, conceptualized the *post-hoc* analyses, and revised the manuscript. All authors contributed to the article and approved the submitted version.

## Conflict of Interest

The authors declare that the research was conducted in the absence of any commercial or financial relationships that could be construed as a potential conflict of interest.
